# Polymorphism rs3828903 within *MICB* Is Associated with Susceptibility to Systemic Lupus Erythematosus in a Northern Han Chinese Population

**DOI:** 10.1155/2016/1343760

**Published:** 2016-06-28

**Authors:** Yue-miao Zhang, Xu-jie Zhou, Fa-juan Cheng, Yuan-yuan Qi, Ping Hou, Ming-hui Zhao, Hong Zhang

**Affiliations:** ^1^Renal Division, Peking University First Hospital, Beijing 100034, China; ^2^Peking University Institute of Nephrology, Beijing 100034, China; ^3^Key Laboratory of Renal Disease, Ministry of Health of China, Beijing 100034, China; ^4^Key Laboratory of Chronic Kidney Disease Prevention and Treatment, Peking University, Ministry of Education, Beijing 100034, China

## Abstract

*Objectives*. The variant rs3828903 within* MICB*, a nonclassical* MHC* class I chain-related gene, was detected to contribute to systemic lupus erythematosus (SLE) in a Caucasian population. This study aimed to investigate the association in a northern Han Chinese population.* Methods*. We recruited 1077 SLE patients and 793 controls for analysis. rs3828903 was genotyped by TaqMan allele discrimination assay. Using the public databases, its functional annotations and gene differential expression analysis of* MICB* were evaluated.* Results*. Significant association between the allele G of rs3828903 and risk susceptibility to SLE was observed after adjusting for sex and age (*P* = 1.87 × 10^−2^).* In silico* analyses predicted a higher affinity to transcription factors for allele G (risk) and* cis*-expression quantitative trait loci (*cis*-eQTL) effects of rs3828903 in multiple tissues (*P* ranging from 2.79 × 10^−6^ to 6.27 × 10^−38^). Furthermore, higher mRNA expressions of* MICB* were observed in B cells, monocytes, and renal biopsies from SLE patients compared to controls.* Conclusion*. An association between rs3828903 and susceptibility to SLE has been detected in a Chinese population. This together with the functional annotations of rs3828903 converts* MICB* into a main candidate in the pathogenesis of SLE.

## 1. Introduction

Systemic lupus erythematosus (SLE) is a complex autoimmune disease characterized by diverse clinical performances and outcomes [1]. Although its exact pathogenesis remains to be unclear, a number of studies have suggested the genetic component in the pathogenesis of SLE [2].

A significant association between major histocompatibility complex (*MHC*) locus and SLE susceptibility has been detected and validated in multiple populations [3–5]. However, compared with the classical* MHC* genes, the data is still limited about the roles of nonconventional* MHC* genes in SLE. A high-density single nucleotide polymorphism (SNP) screening of* MHC* in SLE demonstrated strong evidence for independent susceptibility regions, including rs3828903 within* MICB*, in a Caucasian population [6].* MICB* belongs to a family of genes located in the* MHC* class I region, which encodes a stress-induced molecule involved in both innate and adaptive immunity. Its receptor NKG2D is mostly expressed on all natural killer (NK) cells and on subsets of NKT, CD8+ *αβ*, and *γδ* T cells [7]. The NKG2D/MIC interaction was engaged in the pathogenesis of various autoimmune diseases [8–10] by altering their activity, including SLE.

*MICB* is known to be polymorphic. Significantly, several polymorphisms of* MICB* have been reported to modify the level of gene expression by altering the binding of transcription factors [11], suggesting that a profound dysregulation of* MICB* expression may cause autoreactive T-cell stimulation. This, in turn, underlies relevant differences in the natural immune response against infections or tumor transformation and autoimmune diseases [12]. rs3828903 is a regulatory variant within* MICB*. In spite of the fact that rs3828903 has been reported to be associated with the susceptibility to SLE [6], there is no information about its functionality or expression. Thus, further studies in different populations are warranted to confirm this finding. What is more important is that functional analyses are necessary in order to study the characteristics of rs3828903 and how it may affect the autoimmune response observed in SLE patients.

The present study was conducted to investigate whether there is also an association between* MICB* polymorphism rs3828903 and susceptibility to SLE in a northern Han Chinese population. Furthermore, using the public databases, the functional annotations of rs3828903 and gene differential expression analyses of* MICB* were evaluated.

## 2. Patients and Methods

### 2.1. Study Population

To identify the association of rs3828903 with SLE, a total of 1077 patients with SLE (31.55 ± 12.95 years, 883 females) who were of Han ethnicity living in north of China were enrolled in this study. The controls were 793 geographically and ethnically matched healthy blood donors (29.38 ± 13.15 years, 257 females).

All the patients met the revised SLE criteria of the American College of Rheumatology (ACR) [13]. The study was approved by the Ethic Review Committee of Peking University First Hospital. All subjects gave a written informed consent.

### 2.2. SNP Selection and Genotyping

The SNP rs3828903 within* MICB*, which was reported to be associated with SLE in a Caucasian population [6], was selected for association analysis. It was genotyped using a TaqMan allele discrimination assay (assay ID: AH0JEOO; Applied Biosystems, Foster City, CA, USA) according to the manufacturer's instructions. The primers are as follows: forward 5′-GGTGGGATAGGGTGAGGAGATC-3′ and reverse 5′-GGAAACCATAGCTCCCACAATCTA-3′. The reporter sequences include VIC 5′-CACCACCTCCATTTC-3′ and FAM 5′-ACCACCCCCATTTC-3′.

### 2.3. Computational Assessment of rs3828903

The DNA features and regulatory elements of the regions that contain rs3828903 were identified by searching HaploReg v4.1 database (http://www.broadinstitute.org/mammals/haploreg/haploreg.php) and RegulomeDB database (http://regulome.stanford.edu/). Using HaploReg v4.1 database, the variant effect of rs3828903 on regulatory motifs was quantified as the difference of LOD (alt) − LOD (ref). A negative score suggested a relatively higher affinity for the reference sequence, while a positive score indicated a relatively higher affinity for the alternative. Besides, the* cis*-expression quantitative trait loci (*cis*-eQTL) effect of rs3828903 was summarized.

### 2.4. Gene Differential Expression Analysis of* MICB*

Using the ArrayExpress Archive database (http://www.ebi.ac.uk/arrayexpress/), gene differential expression analyses of* MICB* were checked in immune cell subsets and renal biopsies from SLE patients and healthy controls. In detail, the analysis data was derived from large-scale genome-wide gene expression analyses which were conducted in peripheral blood mononuclear cells (PBMCs) (E-GEOD-50772), B cells (E-GEOD-4588), CD3+ T cells (E-GEOD-13887), CD4+ T cells (E-GEOD-4588), monocytes (E-GEOD-46907), monocytes from healthy donors incubated with SLE sera (E-GEOD-46920), and tubulointerstitial and glomeruli samples (E-GEOD-32591).

### 2.5. Statistical Analyses

Significant deviation from the Hardy-Weinberg equilibrium in the controls (*P* < 0.05) was excluded. Statistical power was estimated using the software Power and Sample Size Calculations Version 3.0 (http://biostat.mc.vanderbilt.edu/PowerSampleSize) with a two-sided type I error rate of 0.05. To assess the possible association of rs3828903 with SLE, the allelic distribution between cases and controls was analyzed using the chi-square test. The odds ratio (OR) was provided with 95% confidence interval (95% CI). The age and sex were adjusted by logistic regression analysis. Quantitative variables with a normal distribution were expressed as means and standard deviations and the independent-samples *t*-test (2 groups) was used for analysis. Statistical analyses were performed with SPSS 16.0 software (SPSS Inc., Chicago, IL). A two-tailed *P* value of less than 0.05 was considered statistically significant.

## 3. Results

### 3.1. Polymorphism rs3828903 Was Significantly Associated with SLE

The call rate for rs3828903 was 99.20% and the SNP was in the Hardy-Weinberg equilibrium in both cases and controls (*P* > 0.05). Taking into account the expected frequency of rs3828903 risk allele G (58.0%) in the general population, the combined set of 1,077 SLE cases and 793 controls provided a power of 96.0% to detect an association between SLE and the variant, with an OR of 1.4 at the 5% significance level.

The frequency of the risk allele G of rs3828903 was significantly higher in SLE patients as compared with healthy controls (62.26% versus 57.25%; OR = 1.23, 95% CI = 1.07 to 1.42, *P* = 4.75 × 10^−3^). And logistic regression analysis adjusting for sex and age also suggested a significant association between rs3828903 and SLE (OR = 1.30, 95% CI = 1.05 to 1.62, *P* = 1.81 × 10^−2^), indicating its potential role in the pathogenesis of SLE.

### 3.2. Various Regulatory Effects of rs3828903 Were Predicted

**Figure 1:**
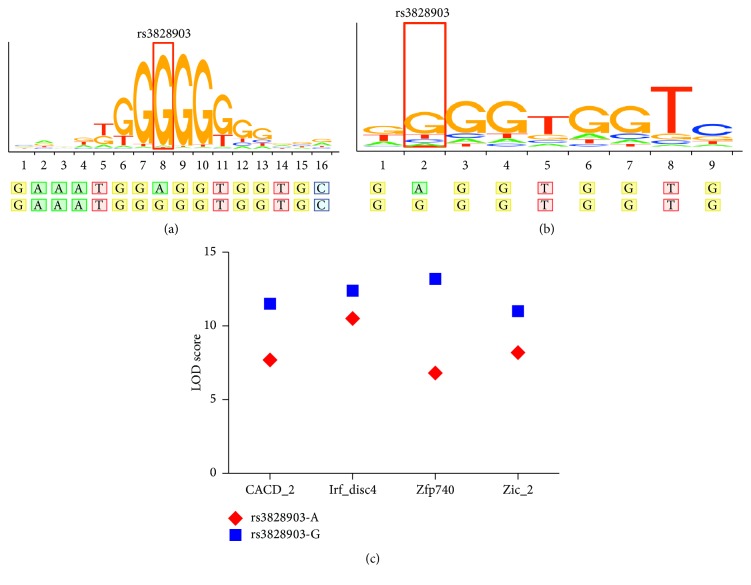
Systemic lupus erythematosus-associated rs3828903 predicted to be part of the motifs for Zfp740 and Zic_2 in both HaploReg v4.1 and RegulomeDB databases. (a, b) Degeneracy within the 16- and 9-base motifs is illustrated at all positions by the stacked letters at each position. The relative height of each letter is proportional to its overenrichment in the motif. A line is boxed around rs3828903-G; this systemic lupus erythematosus-associated risk allele G is predicted to form the 8th and the second nucleotide in the motifs. (c) Altering the rs3828903 allele from rs3828903-G to rs3828903-A decreases the binding affinity for transcription factors CACD_2, Irf_disc4, Zfp740, and Zic_2 in HaploReg v4.1 database.

In HaploReg v4.1 database, rs3828903 was predicted to locate in promoter histone marks, enhancer histone marks, DNase, proteins bound, and motifs changed regions within* MICB*. Four binding site motifs span the rs3828903 region for binding by the transcription factors (TFs) CACD_2, Irf_disc4, Zfp740, and Zic_2. The differences between the LOD scores for the alleles A and G (reference) were −3.8, −1.9, −6.4, and −2.8 for CACD_2, Irf_disc4, Zfp740, and Zic_2, respectively (Figure 1). Therefore, this model predicted a higher affinity to TFs for allele G (risk) relative to allele A. Also, in RegulomeDB database, rs3828903 showed a high score (1f, eQTL + TF binding/DNase peak). Consistent with the HaploReg v4.1 database, the motifs for binding by the TFs Zfp740 and Zic_2 have also been observed in RegulomeDB database (Figure 1), suggesting its potential role for gene expression regulation.

Considering the regulatory effects mentioned above, the* cis*-eQTL effect of rs3828903 has been validated in multiple tissues, including 12 tissues derived from a subset of 1641 samples across 43 sites from 175 individuals and nontransformed peripheral blood samples from 5311 and 1469 unrelated individuals (Table 1). The variant rs3828903 has been detected to affect the expression of* MICB* significantly (with *P* values ranging from 2.79 × 10^−6^ to 6.27 × 10^−38^). Particularly, with an increase in sample size, the association fit was reinforced. This was particularly true for the study, which contained data from 5311 individuals.

### 3.3. Higher Expression Levels of* MICB* Were Observed in SLE

**Figure 2:**
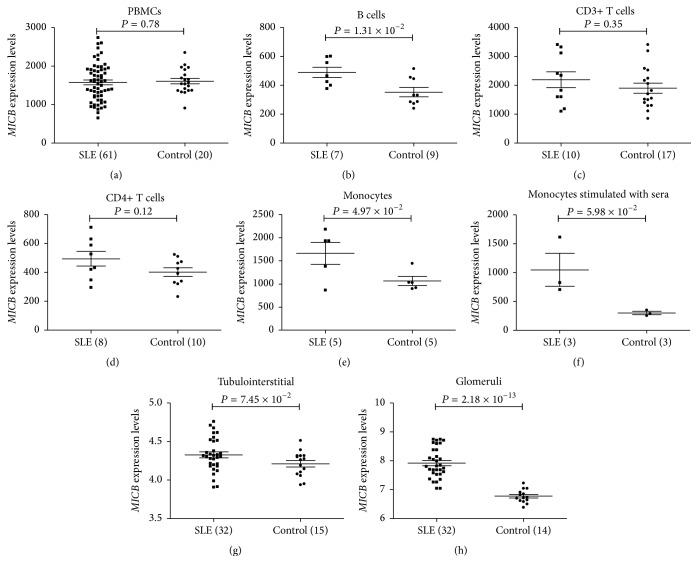
Gene differential expression analyses of* MICB* in immune cell subsets and renal biopsies from SLE patients and controls. (a)–(h) present the* MICB* expression levels in immune cell subsets and renal biopsy samples from SLE patients and normal donor controls.* MICB* mRNA expression was significantly or marginally significantly upregulated in SLE B cells (489.80 ± 95.50 versus 352.66 ± 96.13; *P* = 1.31 × 10^−2^; (b)), monocytes (1661.14 ± 532.87 versus 1065.38 ± 220.72; *P* = 4.97 × 10^−2^; (e)), tubulointerstitial samples (4.33 ± 0.22 versus 4.21 ± 0.16; *P* = 7.45 × 10^−2^; (g)), and glomeruli samples (7.92 ± 0.52 versus 6.77 ± 0.23; *P* = 2.18 × 10^−13^; (h)). Although with a rather small sample size, a marginally significantly higher expression level of* MICB* has been observed in monocytes from healthy donors incubated with SLE sera compared to those incubated with autologous serum (1047.50 ± 494.43 versus 300.40 ± 48.88; *P* = 5.98 × 10^−2^; (f)), while there was no difference of* MICB* mRNA expression in PBMC (1577.45 ± 488.74 versus 1610.67 ± 325.23; *P* = 0.78; (a)), CD3+ T cells (2195.09 ± 865.77 versus 1900.54 ± 715.70; *P* = 0.35; (c)), and CD4+ T cells (495.56 ± 144.59 versus 401.59 ± 94.80; *P* = 0.12; (d)). PBMCs: peripheral blood mononuclear cells; SLE: systemic lupus erythematosus. The expression data of* MICB* was captured from ArrayExpress Archive database (http://www.ebi.ac.uk/arrayexpress/).

Using the ArrayExpress Archive database, we further ascertained whether* MICB* was expressed differently in SLE patients and healthy controls. As was shown in Figure 2,* MICB* mRNA expression was significantly or marginally significantly upregulated in SLE B cells (489.80 ± 95.50 versus 352.66 ± 96.13; *P* = 1.31 × 10^−2^; 7 SLE patients versus 9 controls), monocytes (1661.14 ± 532.87 versus 1065.38 ± 220.72; *P* = 4.97 × 10^−2^; 5 SLE patients versus 5 controls), tubulointerstitial samples (4.33 ± 0.22 versus 4.21 ± 0.16; *P* = 7.45 × 10^−2^; 32 SLE patients versus 15 controls), and glomeruli samples (7.92 ± 0.52 versus 6.77 ± 0.23; *P* = 2.18 × 10^−13^; 32 SLE patients versus 14 controls). Interestingly, although with a rather small sample size, a marginally significantly higher expression level of* MICB* has been observed in monocytes from healthy donors incubated with SLE sera compared to those incubated with autologous serum (1047.50 ± 494.43 versus 300.40 ± 48.88; *P* = 5.98 × 10^−2^; 3 SLE patients versus 3 controls), while there was no difference of* MICB* mRNA expression in PBMC (1577.45 ± 488.74 versus 1610.67 ± 325.23; *P* = 0.78; 61 SLE patients versus 20 controls), CD3+ T cells (2195.09 ± 865.77 versus 1900.54 ± 715.70; *P* = 0.35; 10 SLE patients versus 17 controls), and CD4+ T cells (495.56 ± 144.59 versus 401.59 ± 94.80; *P* = 0.12; 8 SLE patients versus 10 controls) (Figure 2).

## 4. Discussion

In this study, a significant association between G allele of rs3828903 and the risk susceptibility to SLE has been detected. The risk G allele showed a higher affinity to the TFs, which significantly affects the expression level of* MICB*. Accordingly, a significantly higher expression level of* MICB* has been observed in SLE patients compared with controls, suggesting the important role of* MICB* in SLE.

SLE is a complex autoimmune disease with periods of waning disease activity and intermittent flares. Various external factors, such as infection, smoking, and ultraviolet light, were suggested to be involved in the disease pathogenesis. MICB belongs to a “stress-induced” family of MHC I-like proteins, which is generally expressed in normal tissues and monocytes. It can be induced by stress, such as heat shock [14], oxidative stress [15], viral and bacterial infections [16], DNA damage [17], and tumorigenesis [17], acting as danger signals to alert NK cells and the subsets of NKT, CD8+ *αβ*, and *γδ* T cells through engagement of the NKG2D activating receptor [7]. As it has been widely accepted that NKG2D/MIC interaction is essential for NK cells and CD8+ T cells to sense the abnormal cell and subsequently eliminate it, MICB was regarded to play an important role in immune regulation in the pathogenesis of SLE. Epstein-Barr virus (EBV) is one of the most common infections in SLE, and suppression of* MICB* expression is employed by Epstein-Barr virus to escape NK cell recognition [18]. However, in the present study, it is a pity that we had no data about EBV positivity available for our patients cohort. In spite of the fact that no starting assumptions about disease pathogenesis are required except that genetic variation contributes to disease and that, starting from this recognition, the genes that are causally related to disease pathophysiology can be reliably identified, it should be of special interests for future pathogenesis studies to investigate if there are differences about EBV positivity between SLE and control subjects, since a huge amount of the world population is positive for EBV.

In the present study, we observed a significant association between the risk allele G of rs3828903 within* MICB* and SLE. The HaploReg v4.1 and RegulomeDB databases predicted a much higher affinity between the factor-binding site of rs3828903 risk allele G and the TFs, which could be the cause of its higher transcription level of* MICB* found in* in silico* analyses. Moreover, higher expressions in B cells, monocytes, and renal biopsies from SLE patients have been observed which may contribute to disease progression through activating NK cells and costimulating effector T cells. However, the differential gene expression of* MICB* was not observed in PBMC and T-cell subsets from SLE patients, which may be due to the fact that* MICB* was mainly expressed in normal tissues and monocytes.

To conclude, we have found that the allele G of rs3828903 was significantly associated with risk susceptibility to SLE in the current population. These data together with the functional annotations of rs3828903 convert* MICB* into a main candidate for being an additional* MHC* gene associated with SLE susceptibility.

## Figures and Tables

**Table 1:** *cis*-eQTL effect of rs3828903 in multiple tissues in HaploReg v4.1 database.

Study	PMID	Tissue	Number	*P* value
GTEx2015_v6	25954001	Adipose_Subcutaneous	94	6.17 × 10^−18^
GTEx2015_v6	25954001	Artery_Aorta	24	4.81 × 10^−14^
GTEx2015_v6	25954001	Artery_Coronary	9	6.96 × 10^−8^
GTEx2015_v6	25954001	Artery_Tibial	112	3.45 × 10^−17^
GTEx2015_v6	25954001	Breast_Mammary_Tissue	27	1.16 × 10^−9^
GTEx2015_v6	25954001	Cells_Transformed_fibroblasts	14	5.94 × 10^−6^
GTEx2015_v6	25954001	Esophagus_Gastroesophageal_Junction	—	4.67 × 10^−7^
GTEx2015_v6	25954001	Esophagus_Mucosa	18	2.05 × 10^−14^
GTEx2015_v6	25954001	Esophagus_Muscularis	20	2.03 × 10^−8^
GTEx2015_v6	25954001	Heart_Atrial_Appendage	25	2.79 × 10^−6^
GTEx2015_v6	25954001	Nerve_Tibial	88	9.55 × 10^−7^
GTEx2015_v6	25954001	Skin_Sun_Exposed_Lower_leg	96	2.00 × 10^−10^
Westra2013	24013639	Whole_Blood	5311	6.27 × 10^−38^
Fehrmann2011	21829388	Whole_Blood	1469	9.70 × 10^−10^
